# Genomic and Epigenomic Influences on Resilience across Scales: Lessons from the Responses of Fish to Environmental Stressors

**DOI:** 10.1093/icb/icae019

**Published:** 2024-04-17

**Authors:** David C H Metzger, Madison L Earhart, Patricia M Schulte

**Affiliations:** Department of Zoology, University of British Columbia, Vancouver, BC, V6T 1Z4, Canada; Department of Zoology, University of British Columbia, Vancouver, BC, V6T 1Z4, Canada; Department of Zoology, University of British Columbia, Vancouver, BC, V6T 1Z4, Canada

## Abstract

Understanding the factors that influence the resilience of biological systems to environmental change is a pressing concern in the face of increasing human impacts on ecosystems and the organisms that inhabit them. However, most considerations of biological resilience have focused at the community and ecosystem levels, whereas here we discuss how including consideration of processes occurring at lower levels of biological organization may provide insights into factors that influence resilience at higher levels. Specifically, we explore how processes at the genomic and epigenomic levels may cascade up to influence resilience at higher levels. We ask how the concepts of “resistance,” or the capacity of a system to minimize change in response to a disturbance, and “recovery,” or the ability of a system to return to its original state following a disturbance and avoid tipping points and resulting regime shifts, map to these lower levels of biological organization. Overall, we suggest that substantial changes at these lower levels may be required to support resilience at higher levels, using selected examples of genomic and epigenomic responses of fish to climate-change-related stressors such as high temperature and hypoxia at the levels of the genome, epigenome, and organism.

## Introduction

The concept of resilience has emerged as an important theme across multiple disciplines and has particular prominence in conservation research and policymaking ( Martin-Martin-Breen and Anderies 2011; Capdevila et al. 2021). Indeed, it has been argued that understanding resilience will be pivotal for establishing sustainable relationships between humans and ecosystems in our human-dominated world (Cañizares et al. 2021) and for deciphering how ecosystems will respond to the environmental changes that are already affecting biodiversity (Pecl et al. 2017; Freeman et al. 2019; He and Silliman 2019; Albert et al. 2021; Su et al. 2021). However, understanding the mechanisms and properties that determine the resilience of an ecosystem remains a major challenge.

**Fig. 1.**
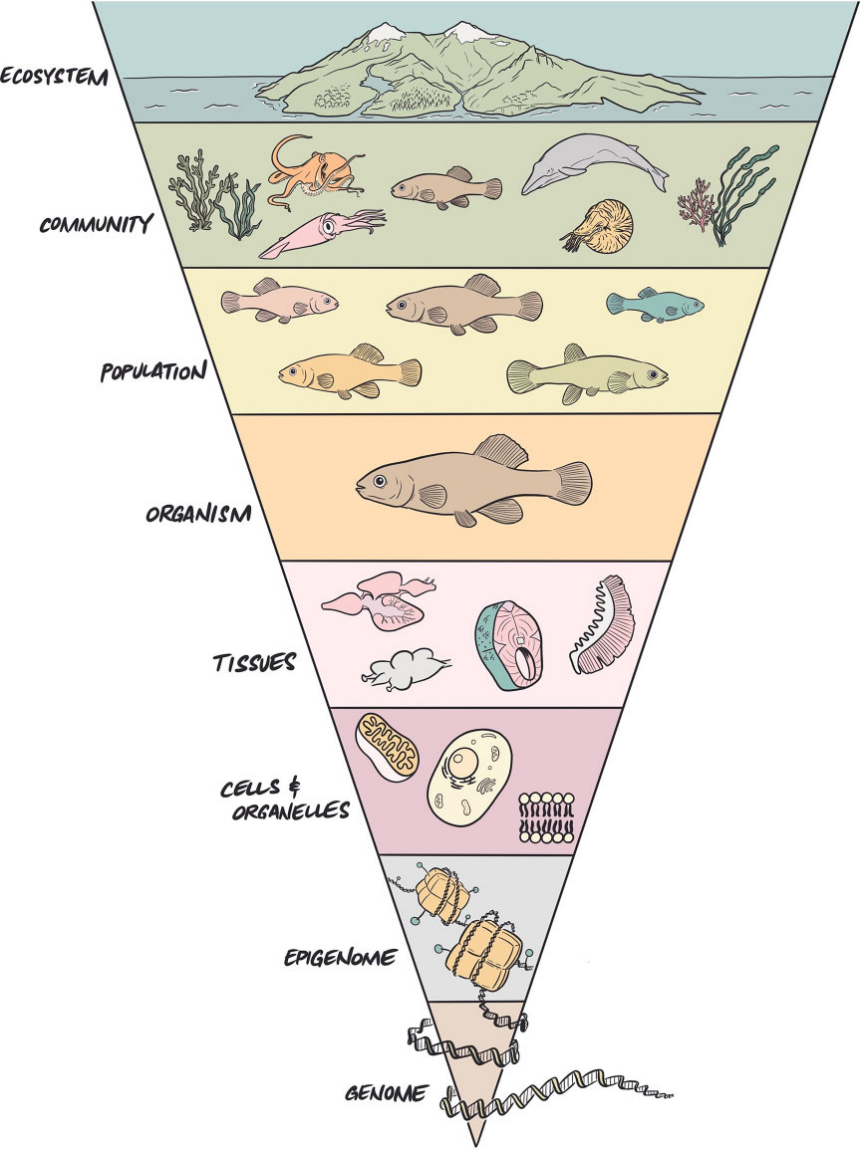
Levels of biological organization. Resilience at the level of communities and ecosystems may be facilitated by multiple interacting processes at lower levels of biological organization. Illustration by Rush Dhillon. **Alt text:** An inverted triangle with different colored sections representing levels of biological organization. The genome is at the bottom (the tip of the inverted triangle) followed by the epigenome, cells and organelles, tissues, organisms, populations, communities, and finally the ecosystem at the top, at the wide end of the triangle.

Here we propose that it may be informative to adopt a framework that acknowledges that resilience at one level of biological organization is likely to be shaped by processes acting at lower levels of organization (Fig. 1). This expansion in focus is driven by the recognition that genomic adaptations, molecular responses, and cellular processes collectively shape the resilience of cells, tissues, and organisms and this is likely to influence the stability and sustainability of populations, communities, and ecosystems (Thorogood et al. 2023). However, despite the extensive consideration of resilience in ecological contexts, how to apply this concept at lower levels of biological organization and what insights might be gained from thinking about resilience at these levels are far from clear.

## Resilience across levels of organization

**Fig. 2.**
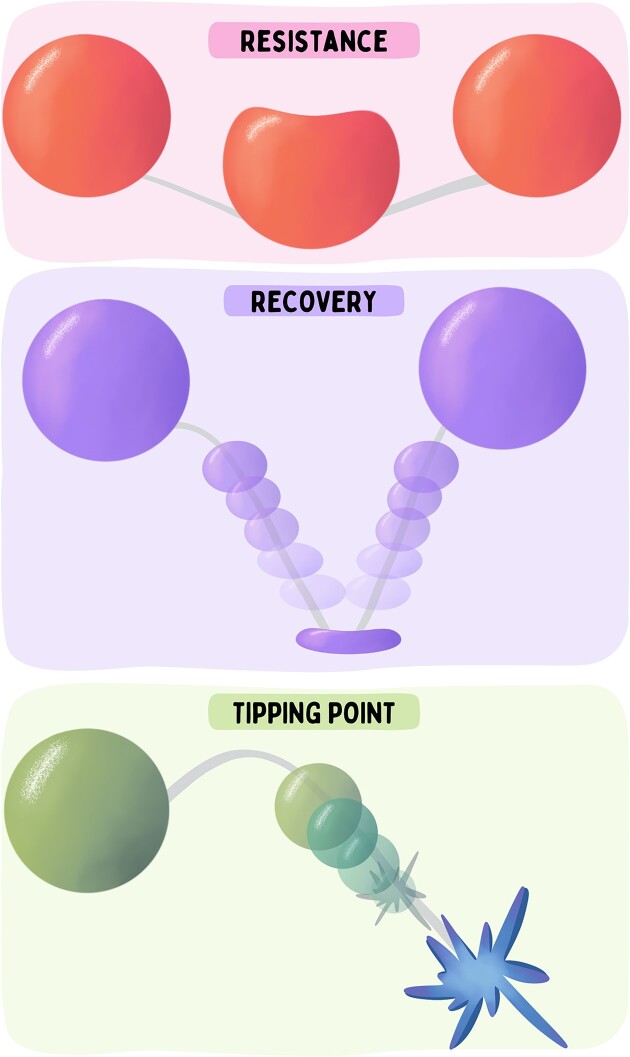
Biological resilience comprises two main components (resistance and recovery). Highly resilient systems may undergo minimal change in response to an environmental stressor (i.e., be resistant) and/or they may have high capacity to recover from the disturbance. When capacities for resistance and recovery are exceeded, systems may reach tipping points at which they transition to a new state. Multiple processes acting within and across biological levels may be important in determining these characteristics. Illustration by Madison Earhart. **Alt text:** Three panels (labeled resistance, recovery, and tipping points) each containing different colored balls. The first panel depicts a red ball getting slightly squished and returning to normal over time. The second panel shows a purple ball bouncing, completely flattening at the bottom, and recovering to normal over time. The third panel depicts a green ball falling over a tipping point and turning into a blue star over time.

The concept of resilience is used in somewhat different ways across disciplines, resulting in multiple calls to refine its definition in the context of the resilience of biological systems (Pimm 1984; Carpenter et al. 2001; Martin-Breen and Anderies 2011; Hodgson et al. 2015; Angeler and Allen 2016; Walker 2020; Capdevila et al. 2021; Yi and Jackson 2021). Many of these definitions embrace two seemingly disparate components of resilience: resistance and recovery (Fig. 2). “Resistance” describes the extent of disturbance a system can endure before reaching a tipping point and shifting to an alternative state, such that a resistant system is able to endure a greater level of a stressor or be disturbed less by a given amount of a stressor (Holling 1973). “Recovery,” on the other hand, reflects the extent to which a system rebounds or recovers following a disturbance (Pimm 1984; Zampieri 2021). “Resistance” and “recovery” may provide alterative or complementary routes to avoiding the shift to an alternative state stable (regime shifts) (Hodgson et al. 2015) (Fig. 2).

Resilience studies often focus on the ability of a community to avoid tipping points to alternative stable states (van Nes et al. 2016; Dakos et al. 2019), presumably because these alternative states are often considered to be undesirable, such as the change from a coral reef-dominated ecosystem to a macroalgal-dominated system (Knowlton 1992). However, in the context of a fundamentally altered environment, a resilient system that is resistant to change may not be the optimal solution as performance may be low in the changed environment. Therefore, a singular focus on resilience in terms of avoidance of tipping points/regime shifts may not always be productive. Instead, it may be necessary to consider how modifying components of the ecosystem could trigger positive tipping points toward new adaptive stable states that maintain ecosystem services (Lenton 2020).

But, how do the concepts of “resistance,” “recovery,” and “tipping points/regime shifts” apply at lower levels of biological organization? In this context, it may be helpful to consider the phenomenon of homeostasis, which is a central unifying principle in physiology (Billman 2020). Homeostasis is the ability of an organism to maintain internal stability in response to environmental change, and represents a form of “resistance” (Fig. 2). However, maintaining homeostasis in any given parameter (e.g., blood pH or body temperature) requires multiple adjustments and changes across processes at lower levels of organization. For example, homeostasis of blood pH in a high CO_2_ environment requires changes in ventilation (at the lungs or gills), alterations of ion transport in tissues such as the gills and kidney, and associated changes in transporter gene expression (Aoi and Marunaka 2014; Hamm et al. 2015; Tresguerres 2024). In essence, homeostasis, and perhaps even biological resilience more generally, may represent examples of the so-called floating duck syndrome, in which substantial changes in underlying mechanisms that are not immediately apparent are required to maintain stability and resilience at higher levels, much like a duck may have to paddle its legs furiously under water to maintain the smooth movement above the water’s surface. Physiological homeostasis and the stability of ecological systems also share similarities in that both are maintained by interactions among multiple feedback systems that provide functional redundancy and increase the stability of the systems through a variety of self-reinforcing feedbacks at lower levels of organization (Baho et al. 2017).

We argue that a variety of responses, or even regime shifts, at lower levels of organization may be key to promoting resilience at higher levels. This idea is exemplified by the phenomenon of phenotypic plasticity, or the ability of an organism to produce different phenotypes in response to changes in environmental conditions without changes in the underlying genotype (Whitman and Agrawal 2009). Although not all phenotypic plasticity results in beneficial changes (Ghalambor et al. 2007), beneficial or adaptive phenotypic plasticity can allow organisms to adjust their morphology, physiology, or behavior to maintain or improve performance when the environment changes. This plasticity at the molecular, cellular, and organismal levels may then promote resilience at higher levels of biological organization (Hendry 2016).

In this review, we consider the idea of resilience and its components of resistance, recovery, and tipping points (Fig. 2) from the perspective of the genome and the epigenome, and their cascading effects across levels of biological organization. We suggest that when thinking about resilience at lower levels of biological organization, it may be useful to add the idea of a “response” along with considerations of resistance, recovery, and regime shifts. To illustrate these ideas, we focus on selected examples from our work on the resilience of fish to key climate-change-related abiotic stressors such as high temperature and low oxygen (hypoxia), to ask how processes at lower levels of organization may contribute to the resilience of organisms, populations, or species, and (by extension) to the properties of the communities and ecosystems they inhabit.

## Genomic contributions to resilience

**Fig. 3.**
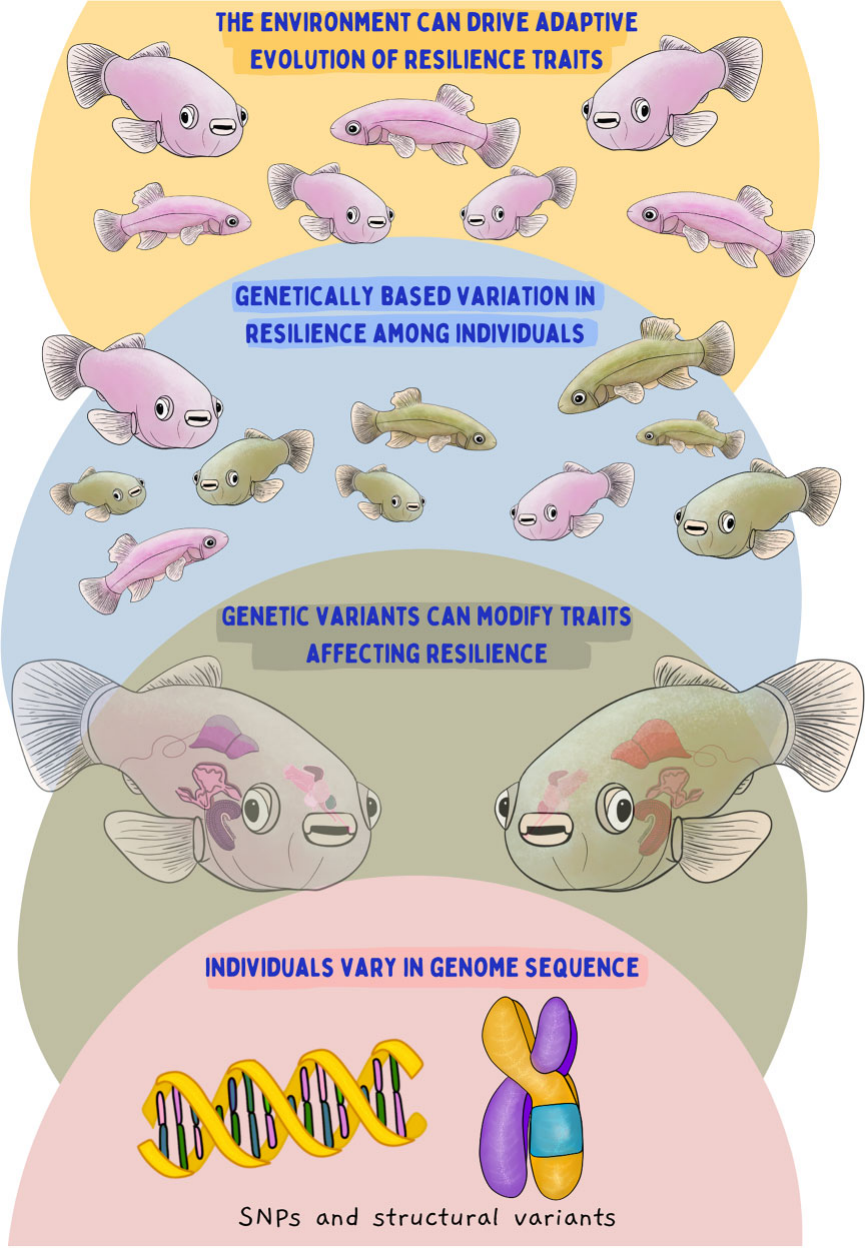
Genomic contributions to resilience. Genome sequences vary within and between species in a variety of ways, including SNPs and structural variants such as insertions, deletions, and rearrangements. Some of these variants may modify traits that affect resilience at the level of individuals and populations. Genetically based variation may increase resilience by buffering responses to environmental disturbance through portfolio effects or by providing standing genetic variation on which natural selection can act. Illustration by Madison Earhart. **Alt text:** Four colored semicircles stacked on top of each other. The bottom semicircle depicts DNA and a chromosome. The next semicircle depicts slightly transparent killifish with different colored organs. The following semicircle includes many killifish, in which some are pink and some are green. The final semicircle depicts multiple killifish again, and this time they are all pink.

The genome encodes the instructions that dictate an organism’s structure, function, and responses to the environment. At the level of the genome itself, molecular processes such as DNA repair, recombination, and replication are central to maintaining genomic integrity (Kim et al. 2019). DNA repair mechanisms, for instance, play a crucial role in fixing errors and damage that occur during replication or as a result of environmental exposures. These processes contribute to the resilience of the genome by ensuring that genetic information remains accurate and functional, and thus the genome is protected by many processes that help it to resist change. However, the genome is also dynamic, undergoing constant change across generations, resulting in genomic diversity within and between species. This diversity is reflected in the presence of single-nucleotide polymorphisms (SNPs) and structural variants, including differences in copy number and genomic rearrangements such as inversions within and between populations and species (Fig. 3).

Genetic variants have the potential to modify traits that are important in responding to environmental stressors, and thus influence the relative resilience of individuals within a population (Schulte and Healy 2022). Increased diversity within and between populations can influence resilience at the population or species levels (Vásquez et al. 2023) through so-called “portfolio effects” (Schindler et al. 2015) because different individuals or populations may respond to environmental change in different ways. This genomic diversity, which represents standing genetic variation, also provides a reservoir of genetic material upon which natural selection can act, allowing species to adapt to diverse ecological niches and respond to changing environmental conditions (Barrett and Schluter 2008). Thus, genetic and functional diversity (the trait variation resulting from this genetic diversity) may be a key component determining resilience at the species and population levels (Barrett and Schluter 2008; Dakos et al. 2019; Schulte and Healy 2022). In addition, it has been argued that both intraspecific diversity in genes encoding key functional traits and the resulting capacity for adaptive evolution may cascade up levels of biological organization to influence ecosystem resilience and susceptibility to tipping points (Dakos et al. 2019). Indeed, the effects of increased intraspecific genetic and functional diversity on community and ecosystem properties have received extensive attention from both theoretical (Norberg et al. 2001) and empirical perspectives (Reusch et al. 2005; Crutsinger et al. 2008; Roger et al. 2012) (for reviews, see Hughes et al. 2008; Bolnick et al. 2011).

Identifying the genetic variants influencing resilience remains a challenging task because resilience is likely to be influenced by a variety of traits, each of which may be highly polygenic (influenced by multiple genes, each with relatively small impacts). Recently, multivariate genome-wide association studies (GWAS), which consider the impacts of multiple loci together, have been successfully used to identify the genetic basis of variation in climate-change-related traits across multiple species (Healy et al. 2018; De La Torre et al. 2021; Yoshida and Yáñez 2021). For example, using a small topminnow that is found in marshes and estuaries along the east coast of North America (the Atlantic killifish, *Fundulus heteroclitus*), we have shown that there is considerable interindividual variation in traits that are thought to be indicative of the ability to resist climate-change-related stressors such as the maximum tolerated temperature (measured as the critical thermal maximum, CTMax) and the minimum tolerated oxygen level (measured as the time to loss of equilibrium in hypoxia, LOEhyp) (Heal et al. 2018). Using a combination of univariate and multivariate GWAS, we demonstrated that these resilience traits have a genetic basis and that genotypes at a relatively small set of SNPs (∼35–40) can account for ∼50% of the phenotypic variation in these traits, with different sets of SNPs being associated with each trait.

Genotype–environment association analyses and genome wide selection scans (GWSS) can also be used to identify genes associated with potential resilience to environmental change (Haasl and Payseur 2016; Lasky et al. 2023). In Atlantic killifish, we have used GWSS (Brennan et al. 2018; Healy et al. 2018) to identify genes whose genetic diversity along a latitudinal thermal gradient has been shaped by selection. By combining the results from GWSS and GWAS, we were able to identify some key candidate genes that are likely to be involved in resilience to high temperature and low oxygen. For example, one of the genes we identified is an E3 ubiquitin ligase, which is one of a large group of genes involved in targeting damaged proteins for degradation (Healy et al. 2018). Ubiquitin ligases are part of the cellular machinery that aids recovery from thermal stress, and thus have a clear link to resilience at the cellular level (Maxwell et al. 2021).

It is also possible to utilize genomic information to make predictions about likely responses to future climate change (Cortés et al. 2020 ). Genomic prediction approaches were first developed to increase the efficiency of plant and animal breeding by predicting phenotypes of particular genotypic combinations, but are now being used to develop predictions for the performance of individuals or populations and their resilience in the face of future climate change (Capblancq et al. 2020; Cortés et al. 2020; Waldvogel et al. 2020). Although many such studies have been conducted in plants (Supple et al. 2018; Cappa et al. 2022), there are only a few compelling studies in wild animals (Capblancq et al. 2020). For example, in yellow warblers (*Setophaga petechia*), associations between genome sequence and climate were used to identify genes linked to exploratory and migratory behavior as potentially important for climate adaptation. Genomic prediction was then used to classify populations as “genetically vulnerable” based on these predictions. The predicted “vulnerable” populations were already showing evidence of declines (Bay et al. 2018). In fish, genomic prediction studies have mostly focused on species that are used in aquaculture or in conservation hatchery stocking programs, because genomic predictions developed using fish in captivity have the potential to predict how wild populations will respond to future environmental change, which may be useful in developing future stocking programs for populations affected by climate change (Yoshida and Yáñez 2021; Sandoval-Castillo et al. 2022).

## Epigenomic contributions to phenotypic plasticity and resilience

Understanding the mechanisms associated with phenotypic plasticity is likely to be a key component of understanding and predicting resilience in the face of environmental change (Williams et al. 2008; Fox et al. 2019; McCaw et al. 2020). In this context, it is critical to appreciate that phenotypic plasticity occurs at a variety of temporal scales, including acute responses (over hours to days), reversible acclimation or acclimatization (over days to months, in the laboratory or the field, respectively; Somero et al. 2017), and developmental plasticity, which occurs when early developmental experience induces phenotypes that may last throughout a lifetime (Whitman and Agrawal 2009). Finally, transgenerational plasticity (TGP) spans multiple generations and occurs when environmental cues induce heritable changes in the phenotypes of offspring (Donelson et al. 2018). Each of these responses to environmental change involves a suite of behavioral, physiological, and molecular responses that, at least in some cases, allow an organism to resist the environmental change and maintain performance.

**Fig. 4.**
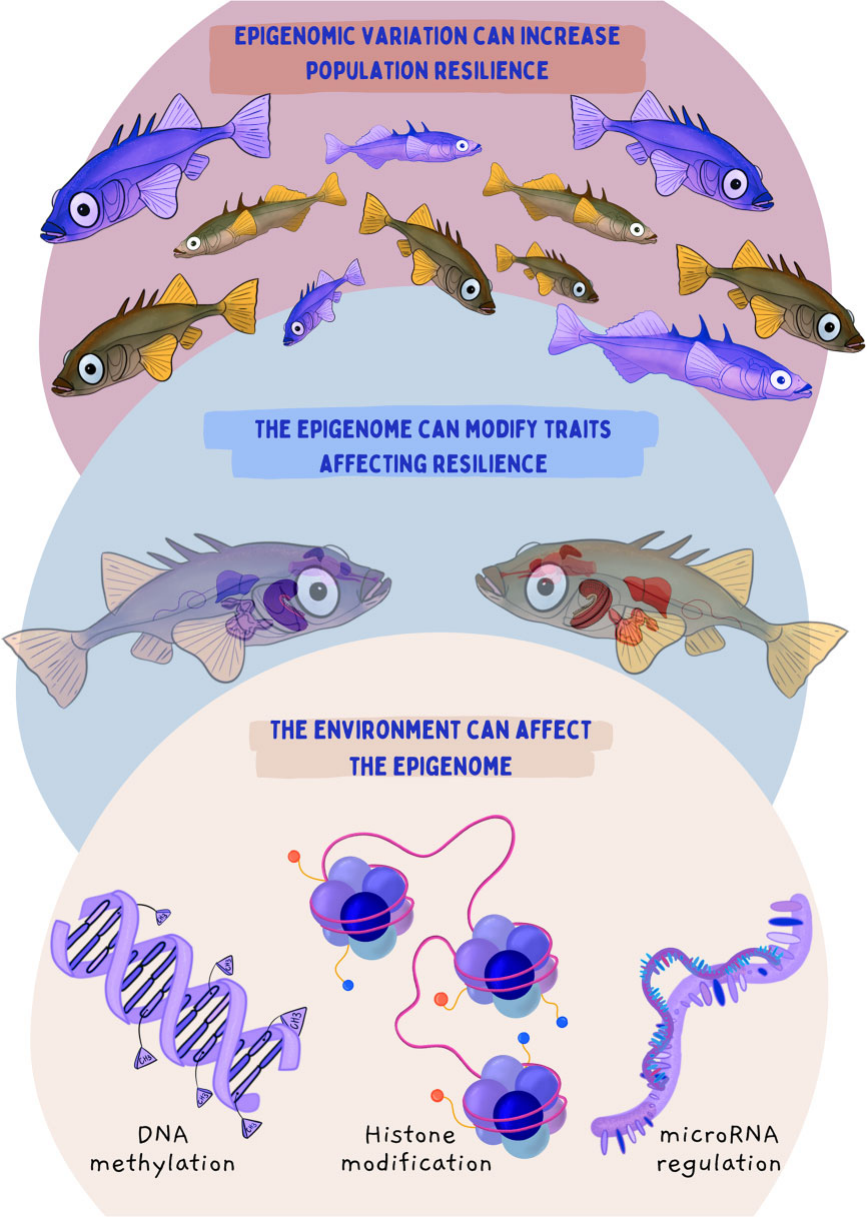
Epigenomic contributions to resilience. Epigenomic mechanisms, including DNA methylation, histone modifications, and regulation of transcription and translation by microRNAs, can modify the activity of the genome without changing the underlying DNA sequence. These epigenomic mechanisms are affected by the environment and thus these mechanisms directly link the genome to environmental change. Changes at the level of the epigenome can affect gene expression, and these effects at the molecular level cascade up to affect traits that may influence organismal resilience to environmental change. This epigenomically mediated variation among individuals or populations can then influence resilience at higher levels of biological organization. Illustration by Madison Earhart. **Alt text:** Three circles stacked vertically. The bottom circle depicts a purple DNA strand with methylation tags, purple histones, and purple micro-RNAs, signifying the epigenome. The middle circle shows two slightly transparent stickleback with either purple or red organs, signifying epigenomic effects on phenotype. The final circle shows a population of stickleback where some individuals are purple and some are normal colored, signifying population diversity in the epigenome.

Epigenetic mechanisms may be critical in determining the capacity for phenotypic plasticity via their role in modulating gene expression (Burggren 2014). Some of the best-characterized epigenetic mechanisms are DNA methylation, histone modifications, and regulation via microRNAs (Fig. 4). Each of these mechanisms has the potential to modify traits that may affect resilience at the organismal level, and (as is the case for genetically based variation) this increased phenotypic diversity may affect resilience at the population and species levels (Schulte and Healy 2022), and cascade upward to affect resilience at community and ecosystem levels (Gladstone-Gallagher et al. 2019).

Two key attributes of epigenetic mechanisms make them intriguing candidates for underlying phenotypic plasticity across timescales: they are environmentally pliable while also being potentially heritable. Thus, epigenetic mechanisms have the capacity to act on short, intragenerational, timescales as well as longer, transgenerational timescales. The ability to dynamically alter the epigenome in response to environmental cues introduces a level of flexibility that is absent in the relatively static nature of DNA. This adaptability allows organisms to make real-time adjustments in gene regulatory processes in response to specific environmental conditions. The potential heritability of epigenetic marks provides a means for adaptive traits to persist in subsequent generations, contributing to the long-term resilience of a species against environmental fluctuations that may ultimately lead to divergence. However, many questions remain before it will be possible to develop a nuanced understanding of the role of epigenomic changes in the context of adaptation and evolution and their importance for determining resilience in the face of climate change (Venney et al. 2023).

Much of the research on the role of epigenomic modification in responses to climate change has focused on DNA methylation, which involves adding or removing a methyl group (CH_3_) to the C-5 position of a cytosine nucleotide in DNA at positions known as CpG sites (Moore et al. 2013). The addition of methyl groups to DNA is catalyzed by members of the *dnmt* (DNA methyltransferase) gene family (Lyko 2018). Various members of the family play different roles. For example, DNMT1 is primarily involved in the maintenance of existing DNA methylation sites, whereas several of the DNMT3s are primarily involved in *de novo* DNA methylation during development and in response to environmental change (Jaenisch and Bird 2003). Thus, viewed through the lens of resilience, some epigenetic patterns are highly robust and resistant to change (maintained by the activity of DNMT1), whereas others are much more dynamically regulated (e.g., through the action of DNMT3s) and can play a role in altering processes of resistance and recovery. Changes in genomic methylation patterns are thought to alter the three-dimensional structure of chromatin, subsequently altering the accessibility of transcription factors and resulting gene expression, affecting traits at higher levels of biological organization (Moore et al. 2013). However, it is important to note that the relationship between DNA methylation patterns and gene expression likely varies among major taxonomic groups (Bogan and Yi 2024). In the vertebrates, genomes tend to be highly methylated, typically with >70% of the CpG sites across the genome being methylated, with a notable exception that actively transcribed genes tend to have low levels of promoter methylation. In contrast, most invertebrate genomes tend to have much lower levels of methylation, with most methylation occurring in gene bodies, and little occurring in promoters or intergenic regions (Suzuki and Bird 2008; de Mendoza et al. 2020). However, in all groups, methylation patterns are sensitive to environmental signals, although the relationship between these changes and changes in gene expression varies among taxa (Bogan and Yi 2024). In particular, in plants and vertebrates, the clear relationship between changes in DNA methylation and gene expression makes this epigenetic mechanism a likely candidate for mediating phenotypic plasticity and determining the capacity for organismal resilience through these phenotypic changes.

**Fig. 5.**
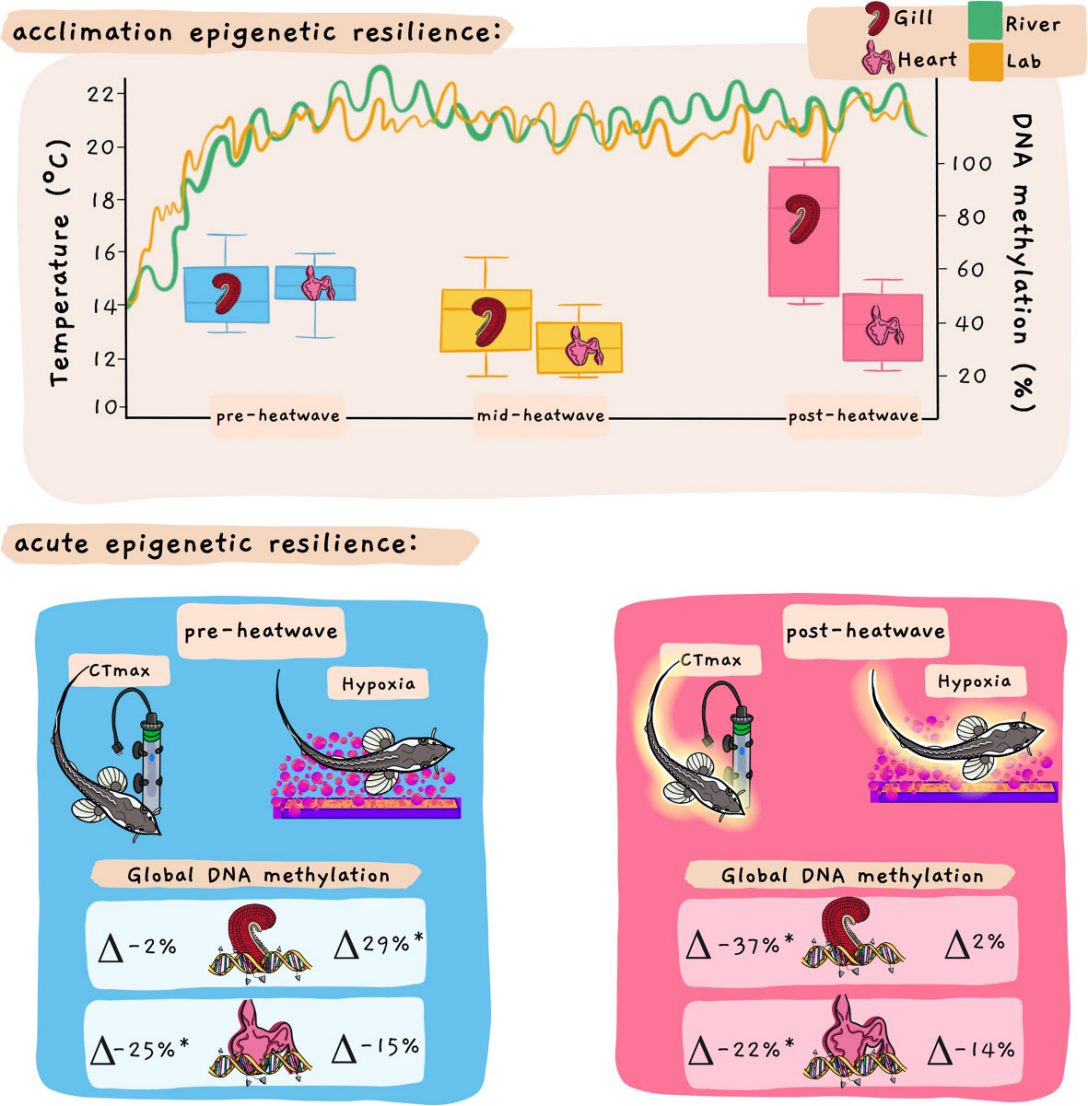
Heatwave acclimation and acute exposure to high temperature or low oxygen alter global levels of DNA methylation in white sturgeon. Juvenile white sturgeon were laboratory acclimated to temperatures mimicking a heatwave recorded in their river of origin across 20 days. Levels of global DNA methylation (%) in the gill and heart changed significantly across the exposure to the simulated heatwave. Heatwave exposure also affected the response of global DNA methylation to acute high-temperature exposure during a CTmax or hypoxia tolerance trial (within ∼1 h). Percent changes in DNA methylation (Δ) relative to levels prior to the tolerance trial are indicated (* indicates statistically significant differences). These responses differ between fish prior to heatwave exposure compared to those that have been exposed to simulated heatwave conditions for 20 days. Data from Earhart et al. (2023). Illustration by Madison Earhart. **Alt text:** An illustration with three rectangles. The top rectangle contains a hand-drawn graph showing river temperature and DNA methylation over time. The two bottom rectangles show illustrations of CTMax and hypoxia trials with juvenile sturgeon next to a water heater and a sturgeon next to a bubble-bar releasing pink nitrogen bubbles. Below the sturgeon there are illustrations of a gill and a heart showing DNA methylation changes.

Most of what is known about the capacity for animals to reshape their methylome comes from studies of environmental acclimation at timescales of weeks to months, with studies in aquatic organisms detecting changes in DNA methylation with acclimation across to salinity (Morán et al. 2013; Artemov et al. 2017; Metzger and Schulte 2018), hypoxia (Beemelmanns et al. 2021), temperature (Han et al. 2016; Sun et al. 2016; Anastasiadi et al. 2017; Metzger and Schulte 2017; Beemelmanns et al. 2021; Lallias et al. 2021; Fellous et al. 2022), and ocean acidification (Putnam et al. 2016; Liew et al. 2018). For example, using threespine stickleback (*Gasterosteus aculeatus*), we have shown that thermal acclimation results in substantial changes in the methylome that were widely distributed across all chromosomes and involve both increased and decreased methylation (Metzger and Schulte 2017). Similarly, in white sturgeon, we have shown that 10 and 20 days of exposure to a thermal stressor that mimicked a natural heatwave resulted in DNA methylation changes in both the gill and the heart (Fig. 5), although the temporal pattern differed between tissues (Earhart et al. 2023). These changes in DNA methylation were associated with increases in upper thermal tolerance and hypoxia tolerance with acclimation, suggesting a potential role in increasing the resilience of this species to subsequent thermal stress.

In contrast to the abundant studies on the effects of acclimation on DNA methylation in animals, much less is known about the effects of acute exposures (<1 day) to climate-change-related stressors. In a study of sea squirts (*Ciona savignyi*), DNA methylation levels were altered after 1 h of acute exposure to high temperatures, 3 h of acute exposure to low salinity, or 6 h of exposure to either low temperature or high salinity (Huang et al. 2017). However, these changes in DNA methylation were transitory with most returning to basal levels within 48 h despite continued presence of the stressor. Similarly, we have shown that white sturgeon (*Acipenser transmontanus*) exhibit rapid changes in DNA methylation (Fig. 5) in response to both thermal and hypoxic stressors (Earhart et al. 2023). In this study, changes in global DNA methylation were observed after ∼1 h exposure to either high temperature or hypoxia, although the patterns differed between tissues and stressors (Earhart et al. 2023). These data demonstrate the capacity for rapid remodeling of the methylome in response to climate-change-related stressors and point to the possible role of these mechanisms in resilience in the face of rapid changes in temperature and oxygenation such as may occur during heatwaves.

A particularly intriguing finding from our study of heatwave exposure in white sturgeon (Earhart et al. 2023) was that there was a difference in the response of the gill methylome to acute high temperature and hypoxia exposure depending on whether or not the fish had undergone acclimation to the heatwave (Fig. 5), although this effect was not observed in the heart. This indicates that prior thermal acclimation influences the acute response of the methylome to more extreme stressors, revealing a previously unexpected interaction between processes acting at these two different timescales. Similarly, other studies have demonstrated how exposure to environmental stressors early in development can have persistent effects on the methylome that influence the response to acute exposures to subsequent stressors later in life (Uren Webster et al. 2018).

There are relatively few examples that have demonstrated the contribution of changes in DNA methylation to the phenomenon of developmental plasticity in fish (Anastasiadi et al. 2021). In threespine stickleback, we have shown that the environmental temperature experienced during embryonic development (from fertilization to hatch) can have effects on DNA methylation patterns that are still apparent in adult fish that have been reared for ∼8 months in a common environment from the time of hatching (Metzger and Schulte 2017), representing a regime shift to a new stable state. Approximately 25% of the differentially methylated regions (DMRs) associated with variation in developmental temperature were also differentially methylated as part of the thermal acclimation response in adults, pointing to at least some overlap between the processes of thermal acclimation and developmental plasticity, but also clearly highlighting the differences between them. An intriguing study in zebrafish (Loughland et al. 2021) demonstrated that knockout of *dnmt*3a (one of the genes responsible for DNA methylation) modifies the thermal performance curve for swimming performance of adult zebrafish and blocks developmental plasticity in this trait. These data strongly suggest that regulated changes in DNA methylation may be required for developmental plasticity.

It has been even more challenging to empirically demonstrate transgenerational inheritance of DNA methylation patterns. Most of the data supporting this phenomenon have been collected in plants, with fewer clear examples in animals (Horsthemke 2018; Anastasiadi, Venney, et al. 2021; Fitz-James and Cavalli 2022). The challenges of assessing transgenerational inheritance of DNA methylation patterns in animals stem from several issues. First, animal germ cells are present throughout life, and thus any exposure of a parent also exposes the next generation and thus experiments must be carried at least to the F2 generation (or the F3 in the case of exposure of pregnant females) to confirm transgenerational inheritance of an epigenetic change. Second, substantial DNA methylation reprogramming occurs during development across deuterostomes (Skvortsova et al. 2018), which decreases the probability that methylation marks will be transferred across generations. However, the extent of DNA methylation reprogramming appears to vary across vertebrates. For example, in mammals, genome-wide DNA demethylation and remethylation is extensive (Reik et al. 2001; Reik 2007), whereas *Xenopus* maintain high levels of genome DNA methylation during early embryonic development (Veenstra and Wolffe 2001). Fish appear to employ diverse reprogramming strategies and it may occur at different developmental time points or in response to environmental change (Fellous et al. 2018, 2022). At least some species, such as zebrafish and medaka, may not undergo reprogramming (Mhanni and McGowan 2004; Fang et al. 2013; Jiang et al. 2013; Potok et al. 2013; Ortega-Recalde et al. 2019; Skvortsova et al. 2019; Ross et al. 2023), or the maternal epigenome may be erased in favor of patterns inherited from the sperm (Jiang et al. 2013; Potok et al. 2013). This opens the possibility of transgenerational inheritance of DNA methylation patterns, but the high prevalence of demethylation during development means that robust empirical evidence should be obtained before strong claims can be made. Finally, most studies examining patterns of DNA methylation and phenotypes at higher levels of organization are fundamentally correlative, and causation is difficult to establish (Duncan et al. 2014).

One potential example of acquired DNA methylation patterns having transgenerational effects has been documented in populations of *Poecilia mexicana* that inhabit sulfide-rich springs (Kelley et al. 2021). In this study, the environmental effects of sulfidic habitats were associated with persistent changes in the DNA methylation patterns of red blood cells. Wild caught pregnant females (F0) were transferred to a nonsulfidic laboratory environment, and many of the DMRs identified between wild-caught adults were also present in F1 and F2 individuals reared in the nonsulfidic laboratory environment. These data suggest that DNA methylation patterns acquired in sulfidic environments resist reprogramming or “recovering” to a nonsulfidic state, suggesting that TGP has resulted in a regime shift toward a new epigenomic state. However, *P. mexicana* are livebearing fish, and because pregnant females were brought back to the laboratory, it cannot be conclusively determined whether the DMRs that persisted are truly heritable or an effect of the sulfidic environment on the germ cells in the F1 generation that would have received sulfide exposure. Nevertheless, these data point to the intriguing possibility that transgenerational inheritance of DNA methylation patterns could be an important component determining the resilience of this species.

Here we have focused on the role of changes in DNA methylation as a potential epigenomic mechanism underlying resilience to environmental stressors, but the association between changes in DNA methylation and changes in gene expression (and thus phenotype) varies substantially among major taxonomic groups (Bogan and Yi 2024). In particular, the evidence of these linkages is strong for plants and mammals, variable in fish, and weak or absent in many invertebrates (Bogan and Yi 2024). On the other hand, there are multiple epigenetic mechanisms, in addition to DNA methylation, that can alter chromatin conformation and gene expression that may play a role in regulating phenotypic plasticity. For example, we have consistently detected changes in the expression of a variety of genes that regulate histone methylation and acetylation in response to thermal acclimation in several species of fish, including salmonids (unpublished data) and stickleback (Metzger and Schulte 2018). Indeed, histone deacetylase activity has been shown to be necessary for thermal acclimation in zebrafish (Seebacher and Simmonds 2019). These studies highlight the potentially important, but understudied, role of multiple epigenetic mechanisms in regulating phenotypic plasticity and underscore their likely role in determining resilience in the face of climate change.

Epigenomic mechanisms clearly play an important role in regulating phenotypic plasticity and affecting the functional traits in many organisms, and (by extension) the persistence of populations in the face of environmental change, but to date there have been few empirical studies linking these mechanisms to processes acting above the species level. However, the evidence linking functional trait diversity within populations to community and ecosystem processes is compelling (Bolnick et al. 2011), which suggests that epigenomic processes that influence functional trait diversity should have similar effects (Hendry 2016). Indeed, there is evidence for community and ecosystem effects of epigenomic variation in plants (Latzel et al. 2013; Rajpal et al. 2022), but whether similar effects occur in animals remains largely unknown.

## Conclusions

We argue that to decipher the critical drivers and indicators of ecosystem-level resilience, it may be important to investigate phenomena acting at lower levels of biological organization. Processes acting at the level of the genome and epigenome can shape responses to environmental change that may affect the capacity for both resistance and recovery at the organismal level, which then have the potential to cascade up to affect resilience at the level of the population, community, or ecosystem. In addition, these genomic and epigenomic processes have the potential to increase levels of physiological diversity among individuals, which allows a form of bet-hedging by providing alternative phenotypes (and genotypes) upon which natural selection can act (Slatkin 1974; O’Dea et al. 2016). Similarly, trait diversity can scale up across biological levels to population and metapopulation structure, and to communities and ecosystems, and this type of functional diversity and potential redundancy has been shown to correlate positively with ecosystem resilience (Mori et al. 2013; Dakos et al. 2019; Biggs et al. 2020). Thus, exploring interindividual genetically or epigenetically based variation in key functional traits has the potential to contribute to our understanding of the resilience of species (and ultimately ecosystems) to environmental change.

## Notes

This paper is a contribution from the SICB 2024 symposium: The scale of resilience: mechanisms of recovery across biological systems.
